# Supplementary material for: Arbitrary engineering of spatial caustics with 3D-printed metasurfaces
Source: Nat Commun. 2024 May 2;15:3719. doi: 10.1038/s41467-024-48026-5 (PMC11065864; doi:10.1038/s41467-024-48026-5)
Supplement: Supplementary file 1 — Supplementary Information [file 41467_2024_48026_MOESM1_ESM.pdf]

Supplementary Information for

Arbitrary Engineering of Spatial Caustics with 3D-printed Metasurfaces

Xiaoyan Zhou[1,2,3], Hongtao Wang[2,3,*], Shuxi Liu[1], Hao Wang[3], John You En Chan[3], Cheng-Feng Pan[2,3], Daomu Zhao[1,*], Joel K. W. Yang[3,*], & Cheng-Wei Qiu[2,*]

Affiliations

[1] Zhejiang Key Laboratory of Micro-nano Quantum Chips and Quantum Control, School of Physics, Zhejiang University, Hangzhou 310058, China

[2] Department of Electrical and Computer Engineering, National University of Singapore, Singapore 117583, Singapore

[3] Engineering Product Development, Singapore University of Technology and Design, Singapore 487372, Singapore

Corresponding author

*E-mail: hongtao_wang@sutd.edu.sg

*E-mail: dmz123@zju.edu.cn

*E-mail: joel_yang@sutd.edu.sg

*E-mail: chengwei.qiu@nus.edu.sg

Inventory of Supplementary Information file:

Supplementary Note 1. The development of optical caustics.
Supplementary Note 2. Derivation of constructing caustics into a spatial focal curve.
Supplementary Note 3. Detailed phase information in Fourier space.
Supplementary Note 4. Detailed theoretical anaylsis of caustics.
Supplementary Note 5. Propagation dynamics of caustic points with a parabolic trajectory.
Supplementary Note 6. Design and optimization of 3D-printed metasurfaces.
Supplementary Note 7. SEM images of nanofins with different heights.
Supplementary Note 8. The effect of rounded surfaces of a nanofin on the optical performance.
Supplementary Note 9. The derivation of higher-order diffractions.
Supplementary Note 10. Derivation of the compensation phase.
Supplementary Note 11. Complex-amplitude information in real space at the initial plane.
Supplementary Note 12. The energy flow of the caustic structured light.
Supplementary Note 13. The effect of fabrication errors on the caustic beams.
Supplementary Note 14. Demonstration about the importance of the compensation phase.
Supplementary Note 15. Z-shaped caustic beams with a parabolic trajectory.
Supplementary Note 16. Geometric-shaped and letter-shaped caustics.
Supplementary Note 17. The propagation-invariant and self-healing features of caustic beams.
Supplementary Note 18. Intensity profiles under the condition of equal radii of Fourier rings.
Supplementary Note 19. Morphed caustics with a linear trajectory.
Supplementary Note 20. Derivation of the amplitude and phase of output from a meta-atom.

Supplementary Note 1. The development of optical caustics.

Research Journey	Ref.	Caustic types in transverse plane	Arbitrary propagation trajectory (Yes or No)	Arbitrarily morphed caustics during propagation (Yes or No)	Wavefront modulation	Optical element / minimum resolution	Applications
Caustics in nature	[1]	Fold	×	×	N.A.	Natural objects (e.g. water, glass, crystals)	Art and decoration
	[2]	Cusp	×	×			
	[3-5]	Seven elementary catastrophes	×	×			
Caustics in structured light	[6]	Fold	×	×	Phase-only	Spatial light modulator / 8 μm	Optical trapping [11]<br>High-resolution microscopy [12]<br>Material processing [13]<br>Communication technology [14]
	[7]	Cusp	×	×			
	[8]	Swallowtail and butterfly	×	×			
	[9]	Umbilic	×	×			
	[10]	Arbitrary structures	×	×			
★ This work		Arbitrary structures	√	√	Complex-amplitude	3D-printed metasurfaces / 1.25 μm	All the applications above will greatly benefit from this arbitrary engineering of caustics.

Tab. S1. Comparison of the researches of optical caustics.

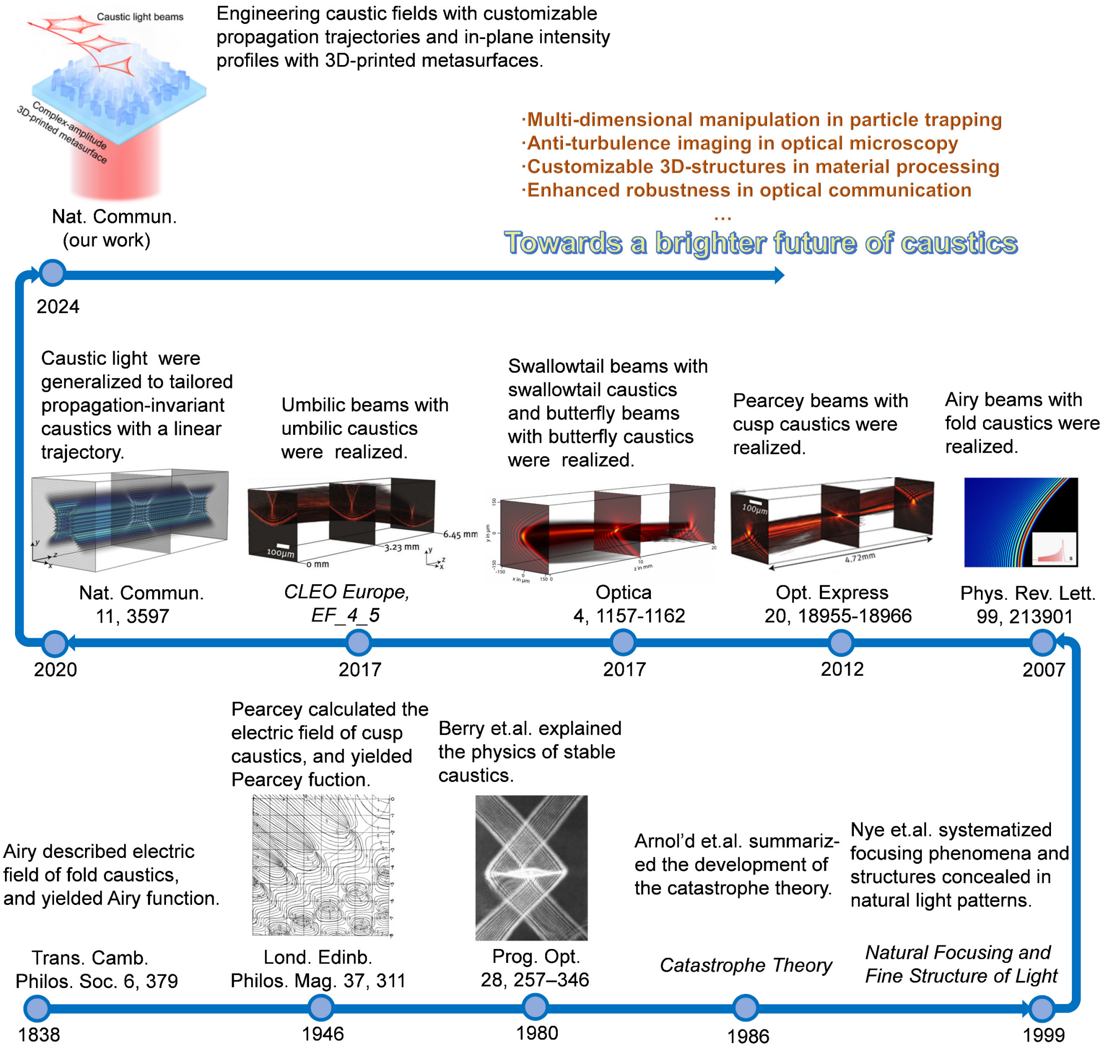

Fig. S1. Roadmap of the development of optical caustics. Reprinted with permission from refs.[2], Taylor & Francis; permission from ref.[3], Elsevier; permission from refs.[6,15], Springer Nature; and permission from ref.[10], CC BY 4.0.

Supplementary Note 2. Derivation of constructing caustics into a spatial focal curve.

We start by employing the angular spectrum method to analyze the propagation of an optical wave along the paraxial *z* direction[16],

$$E(x,y,z)=\exp(ikz)\iint_{-\infty}^{+\infty}F(k_x,k_y)\exp\left[i\left(k_x x+k_y y-\frac{k_x^2+k_y^2}{2k}z\right)\right]\mathrm{d}k_x\mathrm{d}k_y, \tag{1}$$

where (x,y) is an arbitrary position vector in the plane *z*; (k_x,k_y) is corresponding Fourier transform pair in the frequency domain; $k=2\pi/\lambda$ is the wavenumber with λ being the optical wavelength. $F(k_x,k_y)=A(k_x,k_y)\exp\left[i\Phi(k_x,k_y)\right]$ is the initial spectrum distribution with $A(k_x,k_y)$ and $\Phi(k_x,k_y)$ being the amplitude and the phase term, respectively. Based on the method of stationary phase, the significant contribution of the integral arises from critical points of the first kind[1-5], which satisfy

$$\begin{cases}\dfrac{\partial\Psi(k_x,k_y)}{\partial k_x}=\Phi_{k_x}(k_x,k_y)+x-\dfrac{k_x}{k}z=0\\ \dfrac{\partial\Psi(k_x,k_y)}{\partial k_y}=\Phi_{k_y}(k_x,k_y)+y-\dfrac{k_y}{k}z=0\end{cases} \tag{2}$$

with the total phase of the wave component

$$\Psi(k_x,k_y)=\Phi(k_x,k_y)+k_x x+k_y y-\frac{k_x^2+k_y^2}{2k}z. \tag{3}$$

The subscripts k_x and k_y imply the partial derivatives of the corresponding functions. Our task is to determine the phase $\Phi(k_x,k_y)$ in such a way that the caustics of the beams in the transverse plane forms a point-like focus and enables the creation of a spatial focal curve. The parametric functions $X(z)$ and $Y(z)$ govern the trajectory of the beams with the propagation distance *z* acting as a parameter. Thus, we can rewrite Eq. (2) as

$$\begin{cases}\Phi_{k_x}(k_x,k_y)=\dfrac{k_x}{k}z-X(z)\\ \Phi_{k_y}(k_x,k_y)=\dfrac{k_y}{k}z-Y(z)\end{cases} \tag{4}$$

There may be many solutions to this system of equations for a particular $\tilde{z}$, and we assume that they form a continuous locus in the frequency domain, denoted as $C(\tilde{z})$. At this point, we should note that, if any wavevector (k_x,k_y) of its locus $C(\tilde{z})$ is mapped to the distance $\tilde{z}$, then a two-variable function $\tilde{z}(k_x,k_y)$ can be obtained. Moreover, it is crucial to take into account that for $\Phi(k_x,k_y)$ to be twice continuously differentiable, its mixed second-order partial derivatives should be equal, i.e., $\Phi_{k_xk_y}=\Phi_{k_yk_x}$. Therefore, we can deduce the relationship as follows:

$$\tilde{z}_{k_y}\left(\frac{k_x}{k}-X'(\tilde{z})\right)=\tilde{z}_{k_x}\left(\frac{k_y}{k}-Y'(\tilde{z})\right), \tag{5}$$

where the subscripts k_x and k_y refer to the partial derivatives of the corresponding functions; the prime denotes differentiation with respect to $\tilde{z}$. For a certain distance $\tilde{z}$, the functions $X'(\tilde{z})$ and $Y'(\tilde{z})$ are fixed. Thus,

$$\tilde{z}_{kx}\mathrm{d}k_x+\tilde{z}_{k_y}\mathrm{d}k_y=0\,. \tag{6}$$

By combining with Eq. (5), we obtain that

$$(k_x-kX'(\tilde{z}),k_y-kY'(\tilde{z}))\cdot(\mathrm{d}k_x,\mathrm{d}k_y)=0. \tag{7}$$

It is clearly seen that the locus $C(\tilde{z})$ is a circle with its center at $(kX'(\tilde{z}),kY'(\tilde{z}))$, meaning that the wavevectors of the light rays that form a caustic point constitute a circle in the Fourier plane. This also aligns with the physical interpretation of the wavevector of the zero-order Bessel beams: the wavevectors $(k_\perp\cos\varphi,k_\perp\sin\varphi)$ with $k_\perp=\sqrt{k_x{}^2+k_y{}^2}=Cons.$ and φ being the azimuth angle centered at coordinate origin $(0,0)$ constitute the caustic points at arbitrary propagation distance. From this perspective, the movement of the circle's center serves as the fundamental physical mechanism behind the curved propagation trajectory.

Now, the locus $C(\tilde{z})$ can be expressed explicitly as

$$(k_x-kX'(\tilde{z}))^2+(k_y-kY'(\tilde{z}))^2=\beta^2(\tilde{z}), \tag{8}$$

or equivalently,

$$k_x=kX'(\tilde{z})+\beta(\tilde{z})\cos\varphi,\quad k_y=kY'(\tilde{z})+\beta(\tilde{z})\sin\varphi, \tag{9}$$

where $\beta(\tilde{z})$ is the radius. Besides, it is necessary to meet the condition that the distance of the center's movement should not more than the variation in the circle's radius, i.e.,

$$\beta(\tilde{z})=\beta_0\pm nk\int_0^{\tilde{z}}\sqrt{X''(\tilde{z})^2+Y''(\tilde{z})^2}\,\mathrm{d}\tilde{z}, \tag{10}$$

where the constant $|n|\geq 1$. Thus far, taking the partial derivative of Eq. (2) with respect to k_x (or k_y), we arrive at:

$$\frac{\partial\Psi(\tilde{z})}{\partial k_x}=\frac{k\tilde{z}_{k_x}}{2}(X'^2(\tilde{z})+Y'^2(\tilde{z})-\frac{\beta^2(\tilde{z})}{k^2}). \tag{11}$$

At the same time, considering the equation that

$$\frac{\partial\Psi(\tilde{z})}{\partial k_x}=\tilde{z}_{k_x}\frac{\mathrm{d}\Psi(\tilde{z})}{\mathrm{d}\tilde{z}}, \tag{12}$$

we can derive the total phase as

$$\Psi(\tilde{z})=\frac{k}{2}\int_0^{\tilde{z}}\left[\left(\frac{\mathrm{d}X(\xi)}{\mathrm{d}\xi}\right)^2+\left(\frac{\mathrm{d}Y(\xi)}{\mathrm{d}\xi}\right)^2-\left(\frac{\beta(\xi)}{k}\right)^2\right]\mathrm{d}\xi. \tag{13}$$

Here, we set the initial value to zero, since a constant phase difference does not affect the intensity

distribution. Therefore, the phase distribution $\Phi(k_x, k_y)$ can be written as

$$\Phi(k_x, k_y) = \frac{k}{2}\int_0^{\tilde{z}} \left[\left(\frac{\mathrm{d}X(\xi)}{\mathrm{d}\xi} \right)^2 + \left(\frac{\mathrm{d}Y(\xi)}{\mathrm{d}\xi} \right)^2 - \left(\frac{\beta(\xi)}{k} \right)^2 \right] \mathrm{d}\xi - k_x X(\tilde{z}) - k_y Y(\tilde{z}) + \frac{k_x^2 + k_y^2}{2k}\tilde{z}, \qquad (14)$$

where parameter function $\tilde{z}(k_x, k_y)$ is determined by Eq. (8). It is worth mentioning that $0 \le \beta(\tilde{z}) \le k$, which limits maximum propagation distance of the focal curve.

Supplementary Note 3. Detailed phase information in Fourier space.

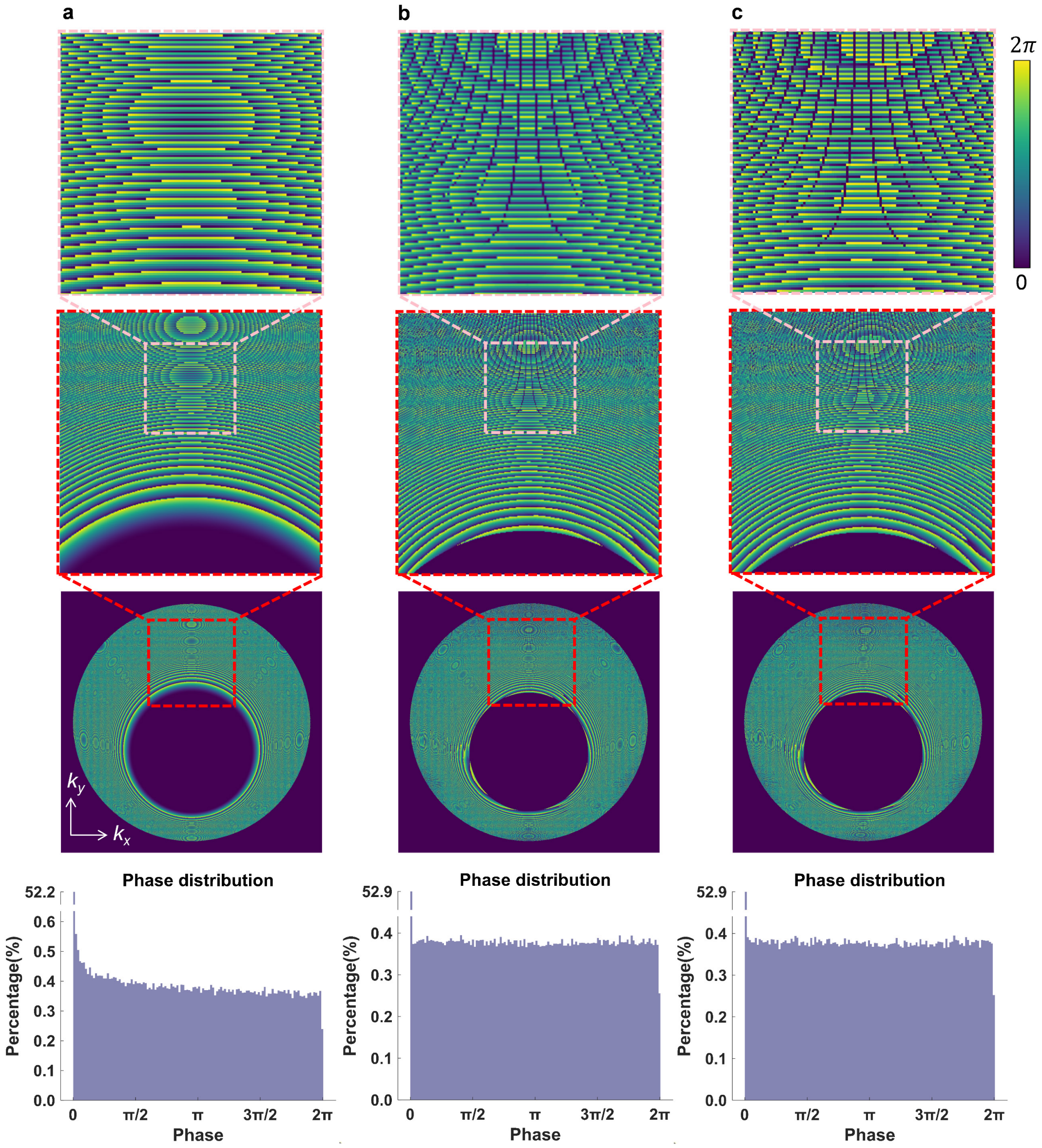

Fig. S2. Phase distributions together with their magnified images and histograms for three cases in the main text.

Supplementary Note 4. Detailed theoretical anaylsis of caustics.

From the perspective of catastrophe theory[17-19], the potential function of the light field, as described by Eq. (1), can be expressed as follows:

$$\mathrm{V} = \Phi(k_x, k_y) + k_x x + k_y y + k_z z - \frac{k_x^2 + k_y^2}{2k} z, \tag{15}$$

The gradient mapping of this potential defines the rays, given by

$$\begin{cases} \dfrac{\partial \mathrm{V}}{\partial k_x} = \Phi_{k_x}(k_x, k_y) + x - \dfrac{k_x}{k} z = 0 \\ \dfrac{\partial \mathrm{V}}{\partial k_y} = \Phi_{k_y}(k_x, k_y) + y - \dfrac{k_y}{k} z = 0 \end{cases}. \tag{16}$$

That implies that, if the point (k_x, k_y, k_z) is selected to satisfy Eq. (16), the normal to the wavefront at (x, y, z) aligns with the direction (k_x, k_y, k_z). The conditions governing the rays establish a mapping between the K-space (k_x, k_y, k_z), called state space, and the observation space (x, y, z), known as control space. For a specific point in state space, one can always find the corresponding point in control space (indicating the ray's direction). However, if given a point in control space, there could be multiple corresponding points in the state space or even none (indicating multiple rays in that specific direction or none at all).

Although a typical point (x, y, z) may correspond to the rays, it does not signify a caustic. To pinpoint a caustic, we must vary (x, y, z) until we reach the envelope of the ray family, which defines the caustic. This process involves ensuring that the potential remains stationary beyond the first order.

Hence, it is necessary to differentiate the ray conditions given by Eq. (16) and determine a set of displacements, denoted as $\mathrm{d}k_x$, $\mathrm{d}k_y$, for which $\mathrm{d}x = \mathrm{d}y = 0$. These can deduce that:

$$\begin{cases} \dfrac{\partial^2 \mathrm{V}}{\partial {k_x}^2} \mathrm{d}k_x + \dfrac{\partial^2 \mathrm{V}}{\partial k_x \partial k_y} \mathrm{d}k_y = 0 \\ \dfrac{\partial^2 \mathrm{V}}{\partial k_x \partial k_y} \mathrm{d}k_x + \dfrac{\partial^2 \mathrm{V}}{\partial {k_x}^2} \mathrm{d}k_y = 0 \end{cases}. \tag{17}$$

The requirement for a solution to exist is that the determinant of coefficients must be zero:

$$\begin{vmatrix} \dfrac{\partial^2 \mathrm{V}}{\partial {k_x}^2} & \dfrac{\partial^2 \mathrm{V}}{\partial k_x \partial k_y} \\ \dfrac{\partial^2 \mathrm{V}}{\partial k_y \partial k_x} & \dfrac{\partial^2 \mathrm{V}}{\partial {k_y}^2} \end{vmatrix} = \begin{vmatrix} \dfrac{\partial^2 \Phi}{\partial {k_x}^2} - \dfrac{z}{k} & \dfrac{\partial^2 \Phi}{\partial k_x \partial k_y} \\ \dfrac{\partial^2 \Phi}{\partial k_y \partial k_x} & \dfrac{\partial^2 \Phi}{\partial {k_y}^2} - \dfrac{z}{k} \end{vmatrix} = 0. \tag{18}$$

Therefore, the caustic, which represents a singularity in the gradient maps, serves as the envelope for a collection of rays.

Supplementary Note 5. Propagation dynamics of caustic points with a parabolic trajectory.

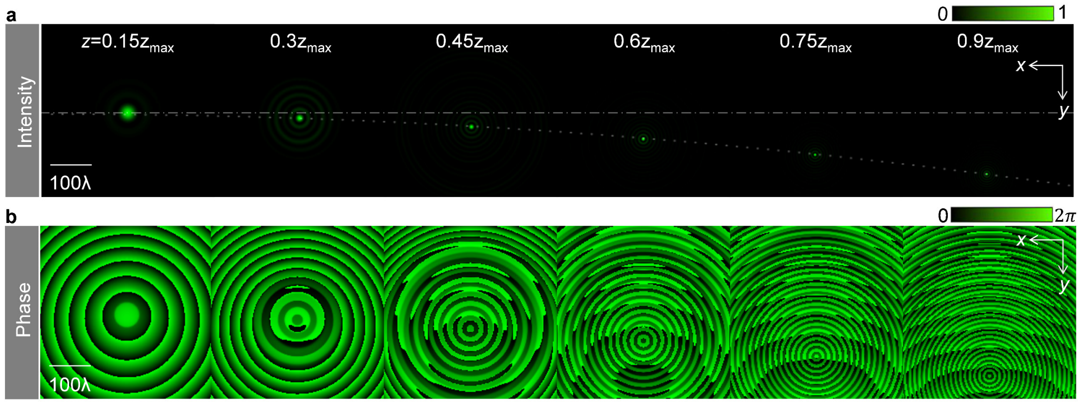

Fig. S3. Propagation dynamics of caustic points with trajectory $X(z)=0$, $Y(z)=2\times10^{-7}z^2/\lambda$. a Simulation results of transverse intensity profiles at propagation distance $z=0.15z_{\max}$, $z=0.3z_{\max}$, $z=0.45z_{\max}$, $z=0.6z_{\max}$, $z=0.75z_{\max}$, and $z=0.9z_{\max}$, respectively. **b** Corresponding transverse phase distributions.

Supplementary Note 6. Design and optimization of 3D-printed metasurfaces.

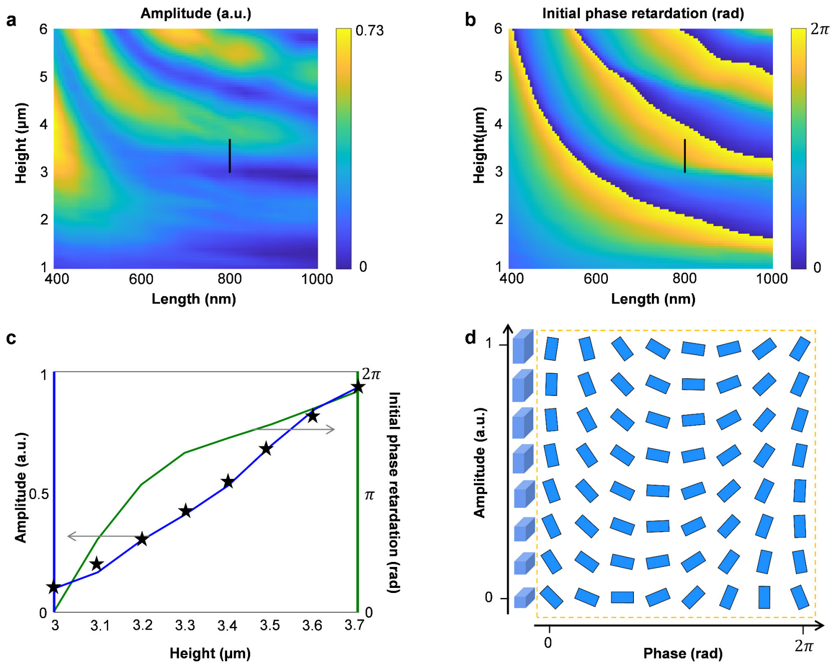

Fig. S4. Design and optimization of 3D-printed metasurfaces for comprehensive and independent control of amplitude and phase responses in transmitted light. a, b Numerical analysis of the amplitude (**a**) and initial phase retardation (**b**) of cross polarization transmitted from a nanofin with different heights (*H*) and lengths (*L*). Vertical black lines in the plots indicate the selection of nanofins for amplitude modulation. **c** Experimental verifications of eight-level amplitude modulation using selected nanofins ($W = 400$ nm and $L = 800$ nm) with different heights at a wavelength of 532 nm. **d** Schematic illustrations of 64-level complex-amplitude modulation based on selected nanofins with eight different heights, each associated with eight distinct in-plane rotation angles.

Supplementary Note 7. SEM images of nanofins with different heights.

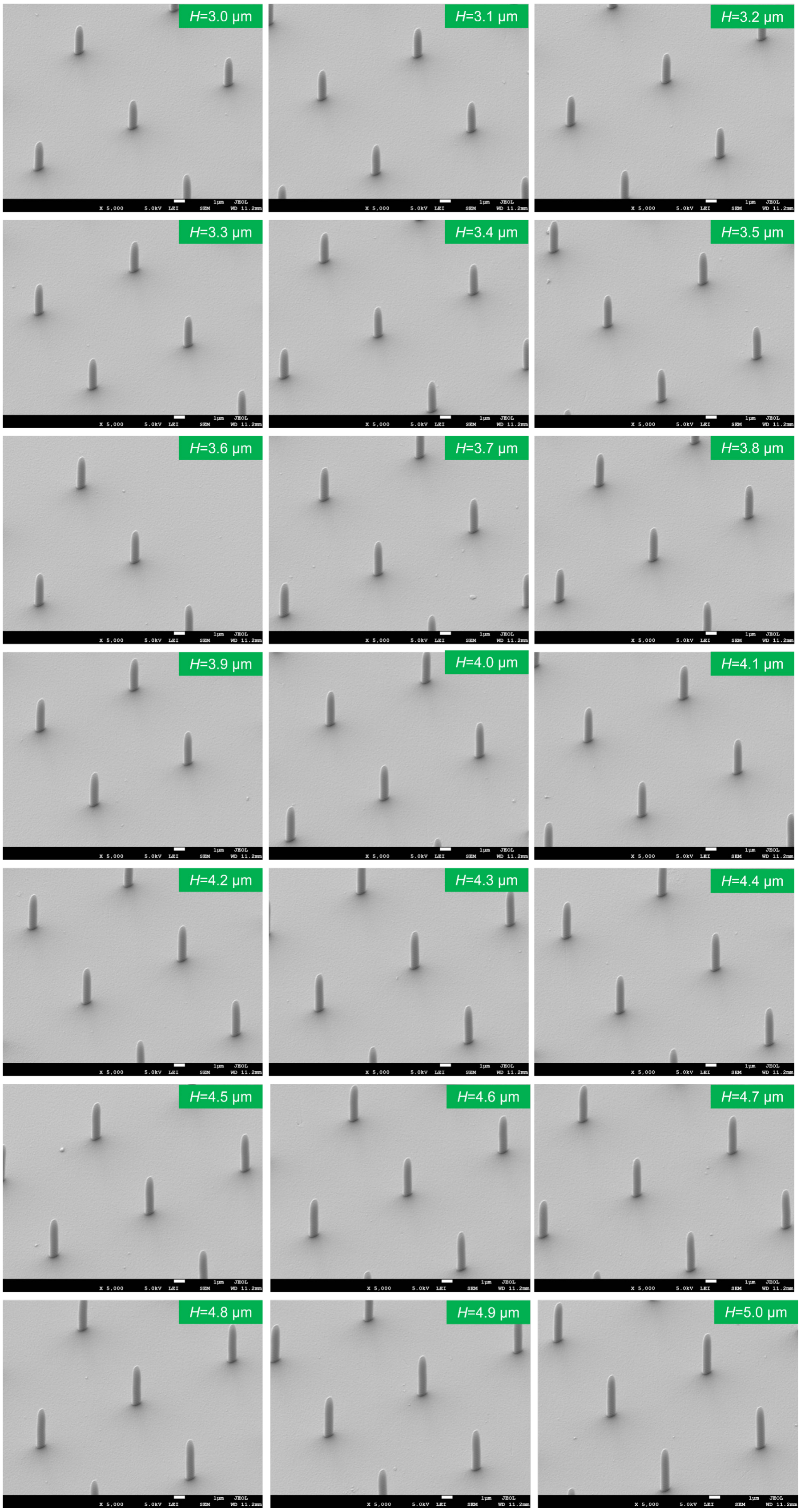

Fig. S5. SEM images of nanofins with heights varying from 3.0 μm to 5.0 μm. All nanofins have uniform transverse dimensions ($W = 400$ nm and $L = 800$ nm).

Supplementary Note 8. The effect of rounded surfaces of a nanofin on the optical performance.

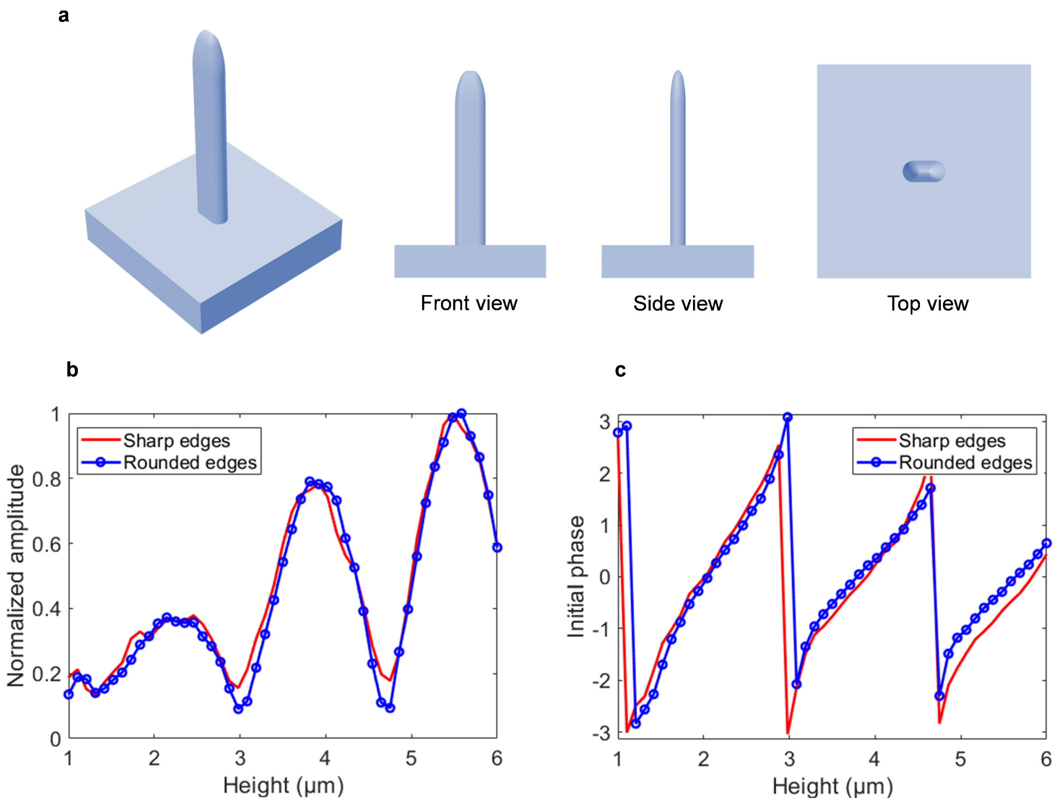

Fig. S6. Simulation analysis of the effect of rounded surfaces of a nanofin on the optical performance. a Schematic of a nanofin with rounded surfaces. **b**, **c** Simulation results of normalized amplitude and initial phase for nanofins with sharp and rounded edges.

Supplementary Note 9. The derivation of higher-order diffractions.

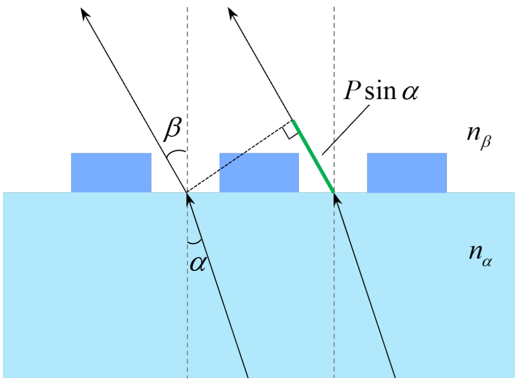

Fig. S7. Cross-sectional schematic of the modeled metasurface.

Figure S7 shows the considered meta-atom, with a nanofin on a glass substrate with refractive index $n_\alpha = 1.5$. The period of the nanofin $P = 1250$ nm. The light wave ($\lambda = 532$ nm) travels from the glass medium to the air with refractive index $n_\beta = 1$. The incident angle is α, and the refraction angle is β. The higher-order diffraction takes place at points of constructive interference, where the criterion is that the difference in optical path length along the two paths equals an integer number of the vacuum wavelength. This condition is expressed as: $m\lambda = P(n_\beta \sin\beta - n_\alpha \sin\alpha)$ with m being the diffraction order. In our case, the incident angle can be regarded as $\alpha = 0$. Thus, only diffraction orders $m = 0$, ± 1, and ± 2 occur. The angle of emergence for the first-order diffraction is $|\beta_1| \approx 25.2°$, and for the second-order diffraction is $|\beta_2| \approx 58.3°$.

Supplementary Note 10. Derivation of the compensation phase.

Let's begin by seeking the compensation phase for the non-diffracting beam propagating along a straight line parallel to the z-axis. We can initiate the derivation from Whittaker's integral[20],

$$\psi(\mathbf{r}) = \int_{-\pi}^{\pi} A(\varphi)\exp[i\Phi(\varphi) + ik_{\perp}\mathbf{r}\cdot\mathbf{u}(\varphi)]d\varphi, \tag{19}$$

where $\mathbf{r} = (x, y)$ is the transverse position vector; $\mathbf{u}(\varphi) = (\cos\varphi, \sin\varphi)$ is a unit vector; $A(\varphi)$ is the amplitude; $\Phi(\varphi)$ is the phase distributions. The caustics can be calculated from the first and second derivatives of the phase of the integral in Eq. (19) with respect to φ with the help of the relationship $\mathbf{r} = (\mathbf{r}\cdot\mathbf{u})\mathbf{u} + (\mathbf{r}\cdot\mathbf{u}')\mathbf{u}'$, which is expressed as

$$\mathbf{r}_c(\varphi) = \frac{1}{k_{\perp}}[\Phi''(\varphi)\mathbf{u}(\varphi) - \Phi'(\varphi)\mathbf{u}'(\varphi)]. \tag{20}$$

By considering the derivative of both sides of the equation, a parametrization of the caustic may be found:

$$\mathbf{r}_c'(\varphi) = \frac{1}{k_{\perp}}[\Phi'''(\varphi) - \Phi'(\varphi)]\mathbf{u}(\varphi). \tag{21}$$

When the caustic is a point located at $\mathbf{r}_c(\varphi) = (x_0, y_0)$, then $\mathbf{r}_c'(\varphi) = 0$, i.e.,

$$\Phi'''(\varphi) - \Phi'(\varphi) = 0. \tag{22}$$

The general solution of this equation is:

$$\Phi(\varphi) = A_1\cos\varphi + A_2\sin\varphi + A_3, \tag{23}$$

A_3 has no physical significance and can be omitted. Thus, Eq. (20) can be rewritten as:

$$\mathbf{r}_c(\varphi) = \frac{1}{k_{\perp}}(-A_1, -A_2). \tag{24}$$

The phase $\Phi(\varphi)$ is finally represented as

$$\Phi(\varphi) = -k_{\perp}\mathbf{r}_c(\varphi)\cdot\mathbf{u}(\varphi). \tag{25}$$

Now, we consider sculpturing the caustic in the form of a desired high-intensity path $\mathbf{r}_p(\tau)$ by using the point caustic we have achieved above. The angular spectrum for such beam is given by

$$\begin{aligned} F(\varphi) &= A(\varphi)\exp[\mathrm{i}\Phi(\varphi)] \\ &= \int_0^l A(\tau)\exp\left[i\phi(\tau) - ik_{\perp}\mathbf{r}_p(\tau)\cdot\mathbf{u}(\varphi)\right]\mathrm{d}\tau, \end{aligned} \tag{26}$$

where l is the overall length of the path; $\phi(\tau)$ is a compensation phase, which is critical to the beam shaping but is yet unknown. By applying the method of stationary phase, the important contribution to Eq. (26) is critical points of the first kind,

$$\frac{\mathrm{d}\phi}{\mathrm{d}\tau} - k_{\perp}\frac{\mathrm{d}\mathbf{r}_p(\tau)}{\mathrm{d}\tau}\cdot\mathbf{u}(\varphi) = 0. \tag{27}$$

This establishes a connection between ray optics and caustics. Specifically, for each τ, there exist certain important rays for the superposition, with wavevectors denoted as $k_{\perp}\mathbf{u}(\varphi)$. Intuitively, these rays should follow the predetermined curve to construct the entire caustic shape. Thus, the unit vector is expressed as

$$\mathbf{u}(\varphi) = \frac{\mathrm{d}\mathbf{r}_p(\tau)/d\tau}{\left|\mathrm{d}\mathbf{r}_p(\tau)/d\tau\right|}. \tag{28}$$

The compensation phase can be calculated as

$$\phi(\tau)=\phi_0+k_\perp\int_0^\tau\left|\frac{\mathrm{d}\mathbf{r}_p(s)}{\mathrm{d}s}\right|\mathrm{d}s, \tag{29}$$

where ϕ_0 is an arbitrary constant term. For simplicity, we set $\phi_0=0$.

On the other hand, the beam's transverse field is given by

$$\begin{aligned}\psi(\mathbf{r})&=\int_0^l A(\tau)\exp\left[i\phi(\tau)\right]\int_{-\pi}^{\pi}\exp\left[ik_\perp(\mathbf{r}_\perp-\mathbf{r}_p(\tau))\cdot\mathbf{u}(\varphi)\right]\mathrm{d}\varphi\mathrm{d}\tau\\&=\int_0^l A(\tau)\exp\left[i\phi(\tau)\right]J_0(k_\perp\left|\mathbf{r}_\perp-\mathbf{r}_p(\tau)\right|)\mathrm{d}\tau,\end{aligned} \tag{30}$$

where $J_0(\cdot)$ is 0th-order Bessel functions. It can be seen very clearly that the electric field in the initial plane is a superposition of 0th-order Bessel functions centered at $\mathbf{r}_p(\tau)=[m(\tau),n(\tau)]^T$ with τ being the length along this path. To ensure the intensity is approximately uniform along the path, we can simply choose $A(\tau)=1/\sqrt{\left|\mathbf{r}_p{}'(\tau)\right|}=1.$ The essence of the problem lies in determining an appropriate value of $\phi(\tau)$ that leads to the field intensity having "maximum" along the path and "minimum" in regions distant from it. $\phi(\tau)=k_\perp\int_0^\tau\left|\frac{\mathrm{d}\mathbf{r}_p(s)}{\mathrm{d}s}\right|\mathrm{d}s$ that we have deduced above is a proper solution to this question.

Now, let's consider the scenario where the beam propagates along a straight line that is not parallel to the *z*-axis. The propagation trajectory is parametrized as (ax,by,z) with *a* and *b* being arbitrary constant. By using the Eq. (9), the angular spectrum for such beam is given by

$$\begin{aligned}F(\varphi)&=A(\varphi)\exp[i\Phi(\varphi)]\\&=\int_0^l A(\tau)\exp\left[i\phi(\tau)-ik_\perp\mathbf{r}_p(\tau)\cdot\mathbf{u}(\varphi)\right]\mathrm{d}\tau\\&=\int_0^l A(\tau)\exp\left[i\phi(\tau)-i\left[(ak+\beta(\tilde{z})\cos\varphi)m(\tau)+(bk+\beta(\tilde{z})\sin\varphi)n(\tau)\right]\right]\mathrm{d}\tau,\end{aligned} \tag{31}$$

where $\phi(\tau)$ represents the compensation phase to be determined. The electric field at plane *z* can be calculated using the method of angular spectrum, written as

$$\psi(x,y,z)=\exp(ikz)\iint_{-\infty}^{+\infty}F(k_x,k_y)\exp\left[i\left(k_xx+k_yy-\frac{k_x^2+k_y^2}{2k}z\right)\right]\mathrm{d}k_x\mathrm{d}k_y. \tag{32}$$

Next, we can deduce to

$$\begin{aligned}\psi(x,y,z)=&\exp\left[i\left(k-\frac{a^2k^2+b^2k^2+\beta^2(\tilde{z})}{2k}\right)z+i\left(akx+bky\right)\right]\\&\int_0^l J_0\left(\beta(\tilde{z})\sqrt{\left(x-az-m(\tau)\right)^2+\left(y-bz-n(\tau)\right)^2}\right)\exp\left[i\phi(\tau)-i\left[akm(\tau)+bkn(\tau)\right]\right]\mathrm{d}\tau.\end{aligned} \tag{33}$$

From the equation, it is evident that the electric field is a superposition of 0th-order Bessel functions $J_0(\cdot)$ centered at $\left(az+m(\tau),bz+n(\tau)\right)$. By analogy with the previous example, the compensation phase can be obtained as follows:

$$\phi(\tau)=\beta(\tilde{z})\int_0^\tau\left|\frac{\mathrm{d}\mathbf{r}_p(s)}{\mathrm{d}s}\right|\mathrm{d}s+akm(\tau)+bkn(\tau). \tag{34}$$

Going even further, we extend the propagation trajectory to arbitrary spatial curve $(X(z),Y(z),z)$,

where functions $X(z)$ and $Y(z)$ respectively govern the trajectory of the beams in x and y directions with the propagation distance z acting as a parameter. The angular spectrum of the beam is represented as

$$F(k_x,k_y)=\int_0^l \exp\left[i\phi(\tau,\tilde{z})+i\Phi_p(\tau,k_x,k_y)\right]\mathrm{d}\tau, \tag{35}$$

where

$$\Phi_p(\tau,k_x,k_y)=\frac{k}{2}\int_0^{\tilde{z}}\left[\left(\frac{\mathrm{d}X(\xi)}{\mathrm{d}\xi}\right)^2+\left(\frac{\mathrm{d}Y(\xi)}{\mathrm{d}\xi}\right)^2-\left(\frac{\beta(\xi)}{k}\right)^2\right]\mathrm{d}\xi-k_x[X(\tilde{z})+m(\tau)]-k_y[Y(\tilde{z})+n(\tau)]+\frac{k_x^2+k_y^2}{2k}\tilde{z}. \tag{36}$$

corresponds to the phase that we have derived above, which allows the trajectory of the light beam to be a curve; $\phi(\tau,\tilde{z})=\phi_1(\tau,\tilde{z})+\phi_2(\tilde{z})$ represents the compensation phase to be determined, which now need be specifically expressed as a function of the length τ and the propagation distance $\tilde{z}$. By substituting Eq. (35) and Eq. (36) into Eq. (32), and considering the relationship expressed in Eq. (9), we can obtain the light field,

$$\psi(x,y,z)=\iiint \begin{matrix}\exp\left\{i\left[\phi_1(\tau,\tilde{z})-kX'(\tilde{z})m(\tau)-kY'(\tilde{z})n(\tau)\right]\right\}\mathrm{d}\tau \\ \beta(\tilde{z})\exp\left\{i\left[kz+\phi_2(\tilde{z})+\Psi(\tilde{z})+\frac{k^2X'^2(\tilde{z})+k^2Y'^2(\tilde{z})+\beta^2(\tilde{z})}{2k}(\tilde{z}-z)+kX'(\tilde{z})\left(x-X(\tilde{z})\right)+kY'(\tilde{z})\left(y-Y(\tilde{z})\right)\right]\right\}\mathrm{d}\tilde{z} \\ \left[\beta'(\tilde{z})+kX''(\tilde{z})\cos\varphi+kY''(\tilde{z})\sin\varphi\right]\exp\left\{i\beta\left[\left(x-X(\tilde{z})-m(\tau)-X'(\tilde{z})(z-\tilde{z})\right)\cos\varphi+\left(y-Y(\tilde{z})-n(\tau)-Y'(\tilde{z})(z-\tilde{z})\right)\sin\varphi\right]\right\}\mathrm{d}\varphi\end{matrix}. \tag{37}$$

It can be observed clearly (regarding the integration with respect to φ): the beam is composed of the superposition of $J_0(\cdot)$, $J_1(\cdot)\cos\varphi$, $J_1(\cdot)\sin\varphi$, with its central position at $\left(X(\tilde{z})+m(\tau)+X'(\tilde{z})(z-\tilde{z}),Y(\tilde{z})+n(\tau)+Y'(\tilde{z})(z-\tilde{z})\right)$. At this point, there are two differences compared to the previous cases.

(1) The optical field is no longer the superposition of only zero-order Bessel beams, but a combination of zero-order and first-order Bessel beams.
(2) The actual superimposed components have a slight deviation from the preset center $\left(X(z)+m(\tau),Y(z)+n(\tau)\right)$. The offset is $\left(X'(\tilde{z})(z-\tilde{z})-\left(X(z)-X(\tilde{z})\right),Y'(\tilde{z})(z-\tilde{z})-\left(Y(z)-Y(\tilde{z})\right)\right)$.

The first issue can be resolved by setting $|n|\gg 1$ in Eq. (10). In this case, the light beam can be approximated as a superposition of zero-order Bessel beams. (In fact, first-order Bessel beams can also achieve similar superposition effects.) The second issue can be corrected by finding a suitable $\phi_2(\tilde{z})$. According to the stationary phase approximation, the $\tilde{z}$ that predominantly contributes to the superposition is determined by the following equation:

$$\begin{aligned}&\frac{\mathrm{d}}{\mathrm{d}\tilde{z}}\left[\phi_2(\tilde{z})+\Psi(\tilde{z})+\frac{k^2X'^2(\tilde{z})+k^2Y'^2(\tilde{z})+\beta^2(\tilde{z})}{2k}(\tilde{z}-z)+kX'\left(x-X(\tilde{z})\right)+kY'\left(y-Y(\tilde{z})\right)\right]\\&=\phi_2'(\tilde{z})+\frac{k^2X'(\tilde{z})X''(\tilde{z})+k^2Y'(\tilde{z})Y''(\tilde{z})+\beta(\tilde{z})\beta'(\tilde{z})}{k}(\tilde{z}-z)+kX''(\tilde{z})\left(x-X(\tilde{z})\right)+kY''(\tilde{z})\left(y-Y(\tilde{z})\right)=0.\end{aligned} \tag{38}$$

By adopting the least squares method, we are supposed to minimize the sum of the squared residuals between z and $\tilde{z}$, when $x=f(z)+m(\tau)$ and $y=g(z)+n(\tau)$. It can be calculated as,

$$\Delta = l\phi_2'^2(\tilde{z}) + 2k\left(X''(\tilde{z})\int_0^l m(\tau)\mathrm{d}\tau + Y''(\tilde{z})\int_0^l n(\tau)\mathrm{d}\tau\right)\phi_2'(\tilde{z})$$
$$+\left(kf''(\tilde{z})\right)^2\int_0^l m^2(\tau)\mathrm{d}\tau + \left(kY''(\tilde{z})\right)^2\int_0^l n^2(\tau)\mathrm{d}\tau + k^2X''(\tilde{z})Y''(\tilde{z})\int_0^l m(\tau)n(\tau)\mathrm{d}\tau. \quad (39)$$

This is a quadratic function with respect to $\phi_2'(\tilde{z})$, and it reaches its minimum value when:

$$\phi_2'(\tilde{z}) = -\frac{2k\left(X''(\tilde{z})\int_0^l m(\tau)\mathrm{d}\tau + Y''(\tilde{z})\int_0^l n(\tau)\mathrm{d}\tau\right)}{2l}$$
$$= -\frac{k}{l}\int_0^l\left[X''(\tilde{z})m(\tau) + Y''(\tilde{z})n(\tau)\right]\mathrm{d}\tau. \quad (40)$$

By integration, we can obtain:

$$\phi_2(\tilde{z}) = -\frac{k}{l}\int_0^l\left[X'(\tilde{z})m(\tau) + Y'(\tilde{z})n(\tau)\right]\mathrm{d}\tau$$
$$= -k\left[X'(\tilde{z})\overline{m(\tau)} + Y'(\tilde{z})\overline{n(\tau)}\right]. \quad (41)$$

After the corrections, we can state that the beam now consists of a superposition of zero-order Bessel functions, and distributes along the desire path. Comparing with the previous example, we can derive that

$$\phi_1(\tilde{z}) = \beta(\tilde{z})\int_0^\tau\left|\frac{\mathrm{d}\mathbf{r}_p(s)}{\mathrm{d}s}\right|\mathrm{d}s + X'(\tilde{z})km(\tau) + Y'(\tilde{z})kn(\tau). \quad (42)$$

Therefore, the total compensation phase is given by:

$$\phi(\tilde{z}) = \beta(\tilde{z})\int_0^\tau\left|\frac{\mathrm{d}\mathbf{r}_p(s)}{\mathrm{d}s}\right|\mathrm{d}s + X'(\tilde{z})k\left[m(\tau) - \overline{m(\tau)}\right] + Y'(\tilde{z})k\left[n(\tau) - \overline{n(\tau)}\right]$$
$$= \beta(\tilde{z})\tau + \mathbf{G}'(\tilde{z})k[\mathbf{r}_p(\tau) - \overline{\mathbf{r}_p(\tau)}]. \quad (43)$$

Here, $\mathbf{G}'(\tilde{z}) = [X'(\tilde{z}), Y'(\tilde{z})]$ is the derivative of the function describing the propagation trajectory; $\mathbf{r}_p(\tau) = [m(\tau), n(\tau)]^T$ represents the transverse path of the caustics; and $\overline{\mathbf{r}_p(\tau)} = [\overline{m(\tau)}, \overline{n(\tau)}]^T$ signifies the center of the path. In a special case when $X(z) = az$ and $Y(z) = bz$, Eq. (43) would accordingly reduce to Eq. (34), differing only by a constant, which has no practical effect on the compensation phase and can be ignored.

Supplementary Note 11. Complex-amplitude information in real space at the initial plane.

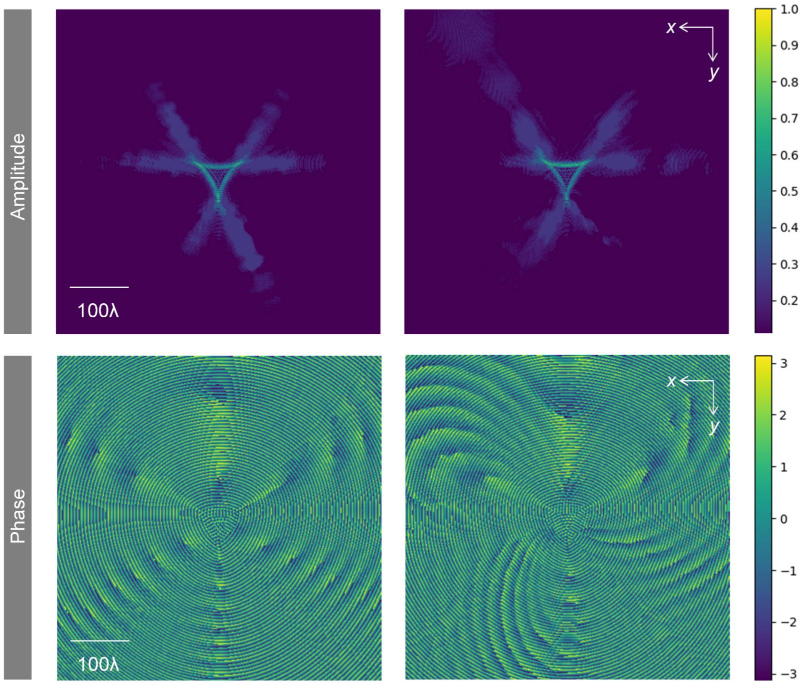

Fig. S8. Complex-amplitude information in real space at the initial plane. The first column corresponds to the case of Fig. 3 in the main text, and the second column corresponds to the scenario in Fig. 4 of the main text.

Supplementary Note 12. The energy flow of the caustic structured light.

The energy flow density vector, also known as the Poynting vector, refers to the amount of energy per unit time flowing through a unit cross section perpendicular to the direction of light propagation [21.22]. In the paraxial regime, the time average of the energy flow density vector is given by,

$$\mathbf{S}=\mathbf{S}_z+\mathbf{S}_\perp=\frac{1}{2\eta_0}|E|^2\,\hat{\mathbf{z}}+\frac{i}{4\eta_0 k}\left(E\nabla_\perp E^*-E^*\nabla_\perp E\right), \tag{44}$$

where $\eta_0=\sqrt{\mu_0/\varepsilon_0}$ is the impedance of free space; $\hat{\mathbf{z}}$ is the unit vector in the *z* direction; $\mathbf{S}_z$ and $\mathbf{S}_\perp$ denote the longitudinal and transverse components, respectively. Among them,

$$\mathbf{S}_\perp=\mathbf{S}_x+\mathbf{S}_y, \tag{45}$$

with

$$\mathbf{S}_x=\frac{i}{4\eta_0 k}\left(E\frac{\partial}{\partial x}E^*-E^*\frac{\partial}{\partial x}E\right) \tag{46}$$

and

$$\mathbf{S}_y=\frac{i}{4\eta_0 k}\left(E\frac{\partial}{\partial y}E^*-E^*\frac{\partial}{\partial y}E\right). \tag{47}$$

We set $\eta_0=377\Omega$, and calculate relative value of the components of the energy flow up light propagation. Figure S9 shows the energy flow distribution of deltoid-shaped caustic beams corresponding to the case illustrated in Fig. 3 in the main text. In this case, we present the energy flow components at propagation distance $z=0.5z_{\max}$. The white arrowheads in the first column denote the magnitude and direction of the transverse energy flow. It can be seen that the energy flow rotates around the deltoid shape. The longitudinal component of the energy flow is always positive, and is much larger than the transverse component (about two orders of magnitude). Therefore, the energy flow rotates and moves in the *y* direction during forward propagation.

Similarly, the energy flow distribution of the morphed caustic beams corresponding to the case illustrated in Fig. 4 of the main text is shown in Fig. S10. Rows 1, 2, and 3 display the transverse and longitudinal components of the energy flow density vector at $z=0.3z_{\max}$, $z=0.6z_{\max}$, and $z=0.9z_{\max}$, respectively. Note that we have shifted the intensity profiles to the middle of the figures for easier observation. In three distinct regions, the transverse energy flow rotates around the shapes of deltoid, astriod, and hypocycloid-5, respectively.

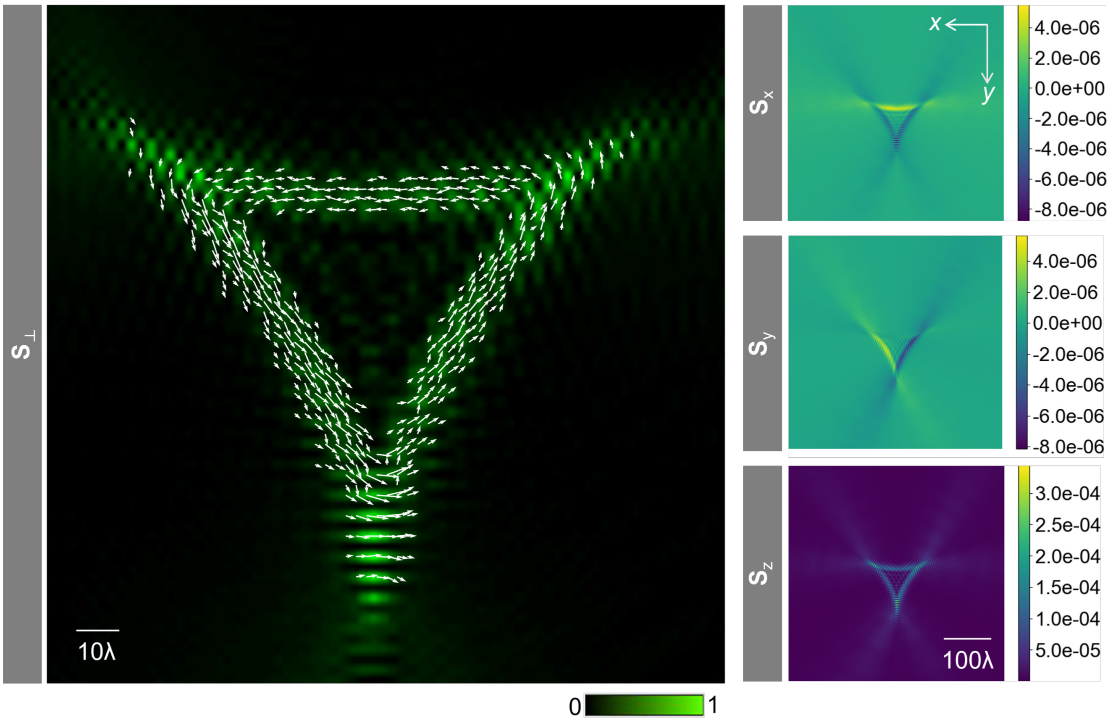

Fig. S9. The energy flow density vector at $z = 0.5z_{\max}$ for the case illustrated in Fig. 3 of the main text. The white arrowheads in the first column denote the distribution of the transverse energy flow.

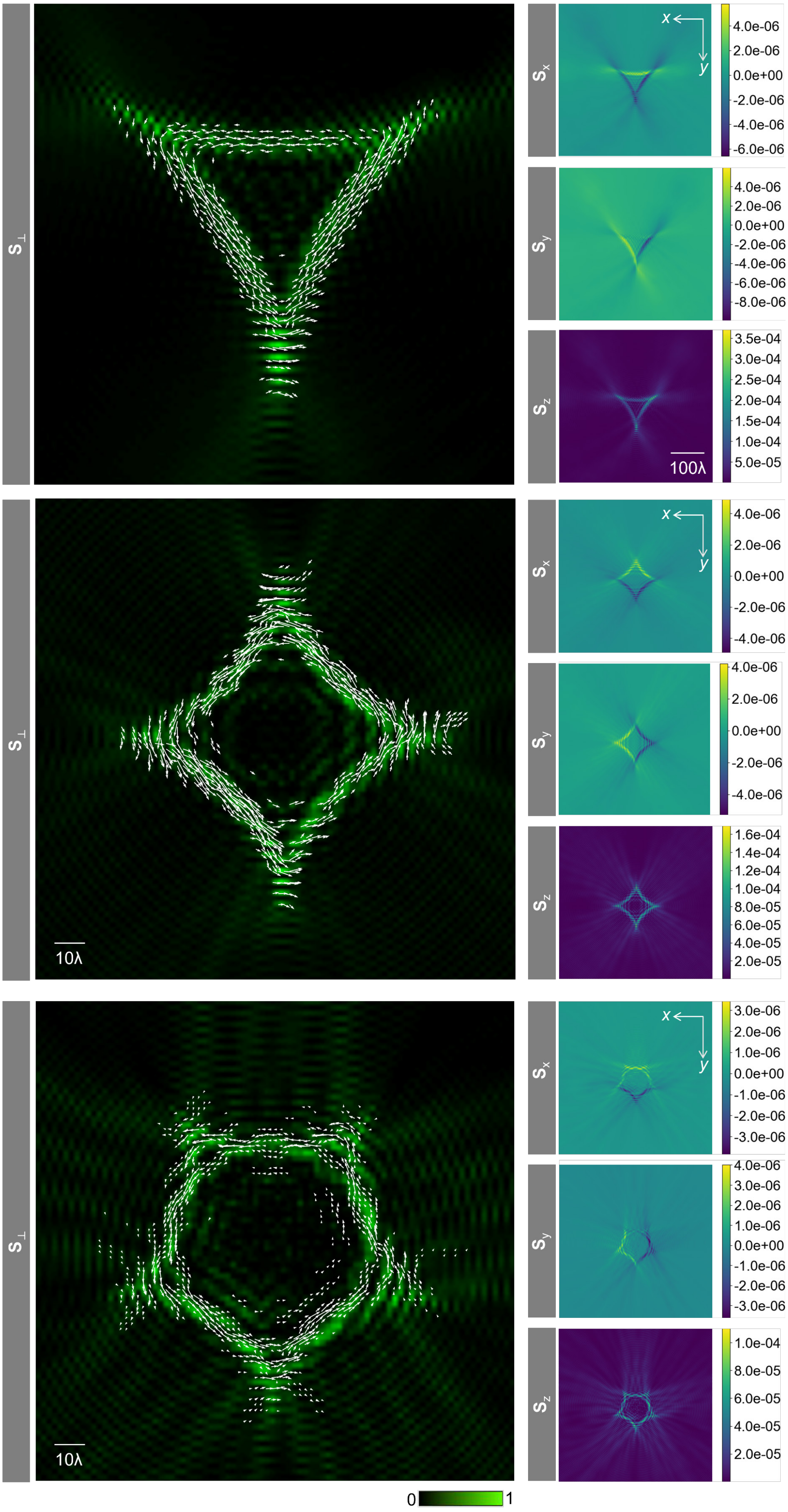
S⊥
Sx
x
y
4.0e-06
2.0e-06
0.0e+00
-2.0e-06
-4.0e-06
-6.0e-06
Sy
4.0e-06
2.0e-06
0.0e+00
-2.0e-06
-4.0e-06
-6.0e-06
-8.0e-06
Sz
3.5e-04
3.0e-04
2.5e-04
2.0e-04
1.5e-04
1.0e-04
5.0e-05
100λ
S⊥
10λ
Sx
x
y
4.0e-06
2.0e-06
0.0e+00
-2.0e-06
-4.0e-06
Sy
4.0e-06
2.0e-06
0.0e+00
-2.0e-06
-4.0e-06
Sz
1.6e-04
1.4e-04
1.2e-04
1.0e-04
8.0e-05
6.0e-05
4.0e-05
2.0e-05
S⊥
10λ
Sx
x
y
3.0e-06
2.0e-06
1.0e-06
0.0e+00
-1.0e-06
-2.0e-06
-3.0e-06
Sy
4.0e-06
3.0e-06
2.0e-06
1.0e-06
0.0e+00
-1.0e-06
-2.0e-06
-3.0e-06
Sz
1.0e-04
8.0e-05
6.0e-05
4.0e-05
2.0e-05
0
1

Fig. S10. The energy flow density vector for the case illustrated in Fig. 4 of the main text. Rows 1, 2, and 3 display the energy flow distributions at propagation distance $z = 0.3z_{\max}$, $z = 0.6z_{\max}$, and $z = 0.9z_{\max}$, respectively. The white arrowheads in the first column denote the distribution of the transverse energy flow.

Supplementary Note 13. The effect of fabrication errors on the caustic beams.

The fabrication error is mainly caused by the machine error, which is a randomly distributed error. Here, we demonstrate the effects of this fabrication error on the caustic structured light. We define the random distribution functions $Random(amplitude)$ and $Random(phase)$ to respectively characterize the amplitude and phase fabrication errors, and use the random factors a and b to respectively measure the random oscillation degree of the amplitude and phase. Thus, the random amplitude is $A_{random} = a \times Random(amplitude)$ and the random phase is $\psi_{random} = b \times Random(phase)$. The total amplitude is $A_{adjusted} = A_0 + A_{random}$ with A_0 being the initial amplitude, and the total phase is $\psi_{adjusted} = \psi_0 + \psi_{random}$ with ψ_0 being the initial phase. We present two different random factors of deltoid-shaped caustic beams in Fig. S11. It can be found that the larger the random factor, the blurrier the beams and the worse the accuracy.

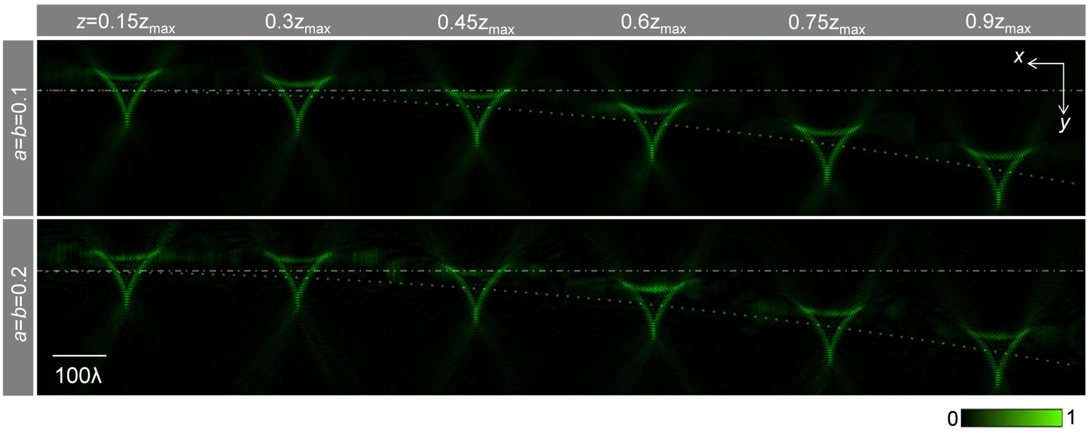

Fig. S11. The effect of fabrication errors with different degrees on the deltoid-shaped caustic beams.

Supplementary Note 14. Demonstration about the importance of the compensation phase.

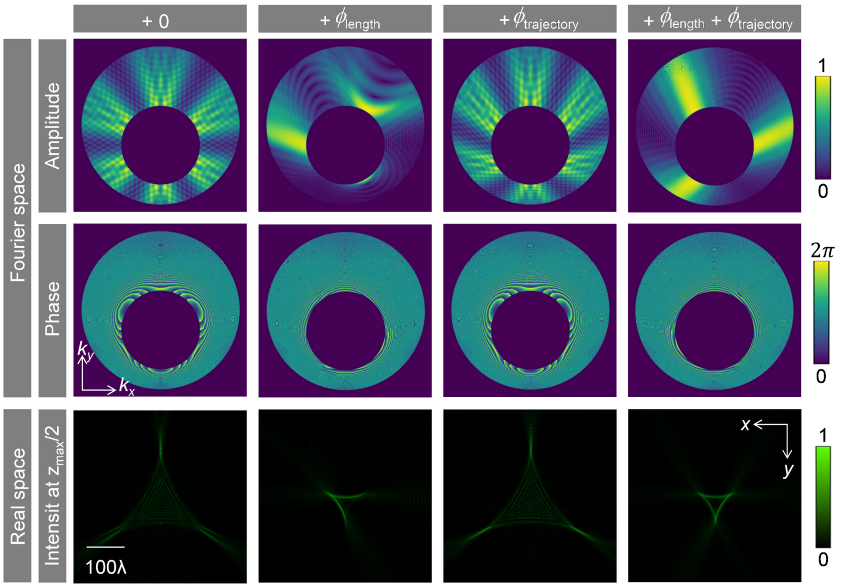

Fig. S12. Complex-amplitude distributions in Fourier space and corresponding real space transverse intensities when different compensation phases are introduced for the case depicted in Fig. 3 of the main text. Rows 1 and 2 depict the amplitude and phase distributions, while row 3 shows the transverse intensity at a specific propagation distance $z = z_{\max} / 2$.

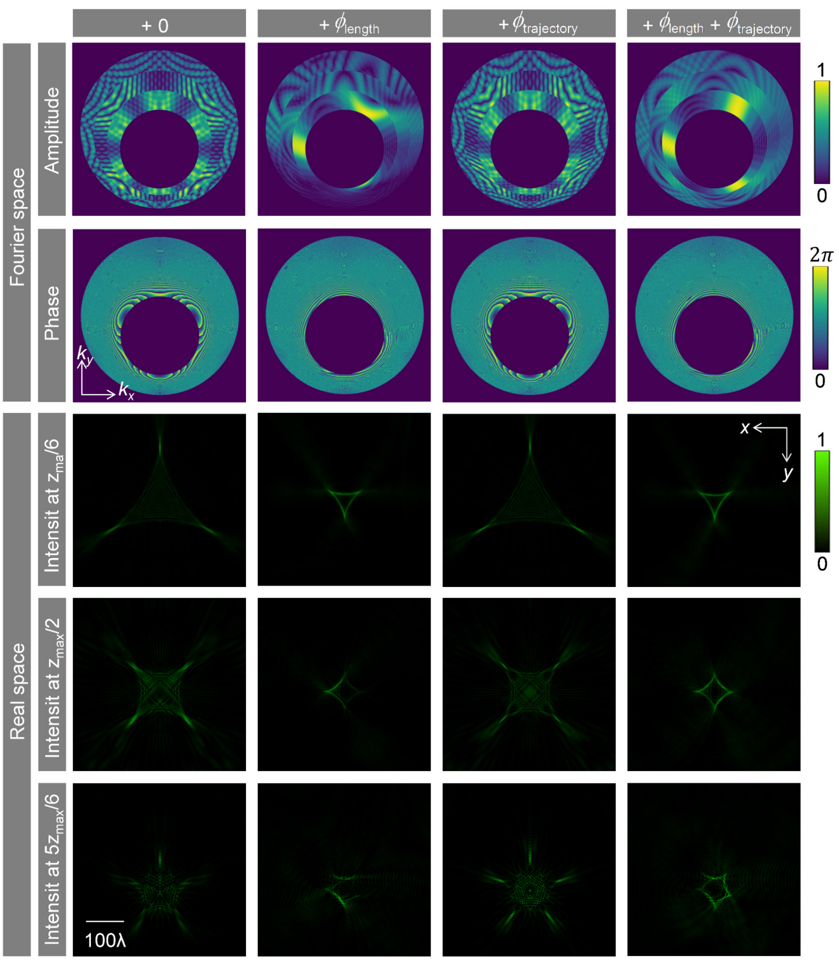

Fig. S13. Complex-amplitude distributions in Fourier space and corresponding transverse intensities in real space when different compensation phases are introduced for the case illustrated in Fig. 4 of the main text. Rows 1 and 2 present the amplitude and phase distributions, while rows 3, 4, and 5 display the transverse intensities at propagation distance $z = z_{\max} / 6$, $z = z_{\max} / 2$, and $z = 5z_{\max} / 6$.

Supplementary Note 15. Z-shaped caustic beams with a parabolic trajectory.

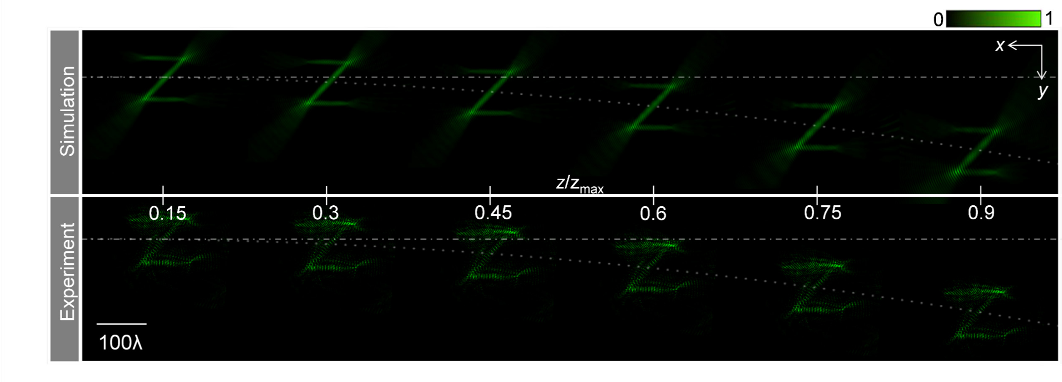

Fig. S14. Simulation and experimental results of Z-shaped caustic beams with a parabolic trajectory.

Supplementary Note 16. Geometric-shaped and letter-shaped caustics.

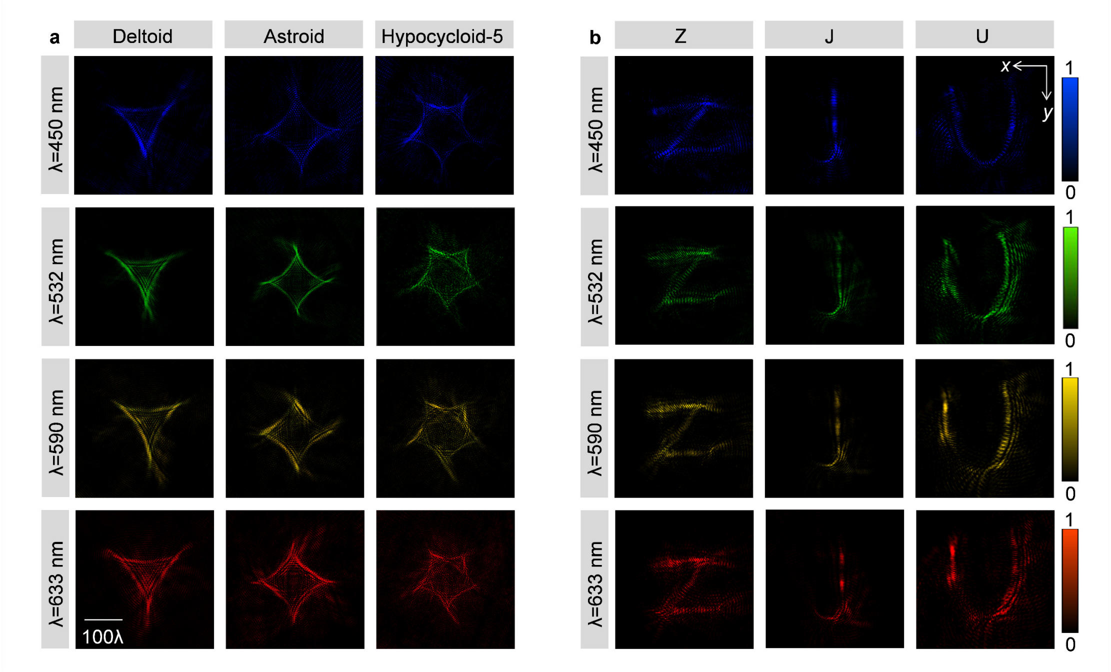

Fig. S15. The measured intensity profiles of desired caustic fields in *x-y* plane at wavelengths of 490 nm (blue), 532 nm (green), 590 nm (yellow) and 633 nm (red). a Geometric-shaped caustics: deltoid, astroid, and hypocycloid with 5 cusps. **b** Letter-shaped caustics: Z, J, and U.

Supplementary Note 17. The propagation-invariant and self-healing features of caustic beams. To demonstrate the propagation-invariant feature of the caustic beams, we need to quantify the similarity of the transverse intensity distributions $I_0(x,y)$ at the reference plane with transverse intensity distributions $I_z(x,y)$ at any longitudinal position *z*. An effective approach is to calculate the normalized cross-correlation function of the intensity distribution patterns[23]:

$$\gamma(m,n)=\frac{\sum_{x,y}\left(I_0(x,y)-\overline{I}_0\right)\cdot\left(I_z(x-m,y-n)-\overline{I}_z\right)}{\sqrt{\sum_{x,y}\left(I_0(x,y)-\overline{I}_0\right)^2\sum_{x,y}\left(I_z(x-m,y-n)-\overline{I}_z\right)^2}}. \tag{48}$$

$\overline{I}_0$ and $\overline{I}_z$ are the average values of I_0 and I_z, respectively. (m,n) is the offset when the two intensity matrices are not aligned. If these two matrices are aligned and equal in size, we can set $m=n=0$. Thus, Eq. (48) is simplified to

$$\gamma=\frac{\sum_{x,y}\left(I_0(x,y)-\overline{I}_0\right)\cdot\left(I_z(x,y)-\overline{I}_z\right)}{\sqrt{\sum_{x,y}\left(I_0(x,y)-\overline{I}_0\right)^2\sum_{i,j}\left(I_z(x,y)-\overline{I}_z\right)^2}}=\frac{cov(I_0,I_z)}{\sigma_{I_0}\sigma_{I_z}}, \tag{49}$$

where $cov(\cdot)$ is the covariance between I_0 and I_z; σ is the standard deviation.

Here, we calculate *z*-dependent normalized cross-correlation for four cases of caustic structured light (deltoid, astroid, hypocycloid-5, and line segment) with a parabolic trajectory, as shown in Fig. S16. The trajectory equation and maximum propagation distance are the same as in Fig. 2 of the main text. The reference plane is chosen as the middle position. Our analysis reveals that within the specified range of propagation distances, the intensity cross-correlation coefficients of the caustic beams predominantly exceed 50%. Further calculations yield average cross-correlation coefficients of these caustic beams with various shapes: 73.8% for deltoid, 63.1% for astroid, 56.0% for hypocycloid-5, and 65.6% for line segment. It is found that all correlation coefficients are greater than 50%, indicating a relatively strong correlation between the fields, and the caustic beam is resistant to diffractive effects.

The field distribution of beams may be perturbed by obstacles or turbulence. Self-healing refers to the process of reconstructing the original structure after such disturbances. Here, we show the self-healing property of the caustic structured light by placing an obstacle in its propagation path. Figure S17 shows the simulation results of blocking a rectangular area (indicated by the white square box in the first column) in the plane $z=0.5z_{\max}$ of the caustic structured light. One corner of the caustic pattern is obscured. During the propagation process, the caustic structure damaged by a partial obstruction gradually recovers. At the distance of $z=0.58z_{\max}$, four types of structures are almost completely repaired. In order to describe the degree of self-healing of the beams detailly, we calculated the normalized cross-correlation coefficients between the deltoid-shaped caustic beams blocked by square obstacles with sides $L=40\lambda$, 60λ, and 80λ, respectively, and the unblocked beams at different propagation distances, together with the intensity distributions at $0.5z_{\max}$ and $0.85z_{\max}$, as shown in Fig. S18. The white square box indicates the obstructed area. From the quantitative calculation, we can find that the normalized correlation coefficient appears a slight oscillation in the early stage, but when the propagation distance is far enough, it becomes higher and higher and finally tends to a stable value. This suggests that the influence of the obstacle is getting weaker and the lateral light intensity distribution is getting more similar. Moreover, the larger the obstacle size, the smaller the correlation coefficient, the slower the self-healing process, and the

longer the recovery distance required. These presented results demonstrate that the caustic beams possess self-healing properties. In the aspect of optical caustics, self-healing after such distortions can be attributed to a reconstruction by rays with a higher inclination that passes the obstacles and contributes to the light field sufficiently far behind the perturbation.

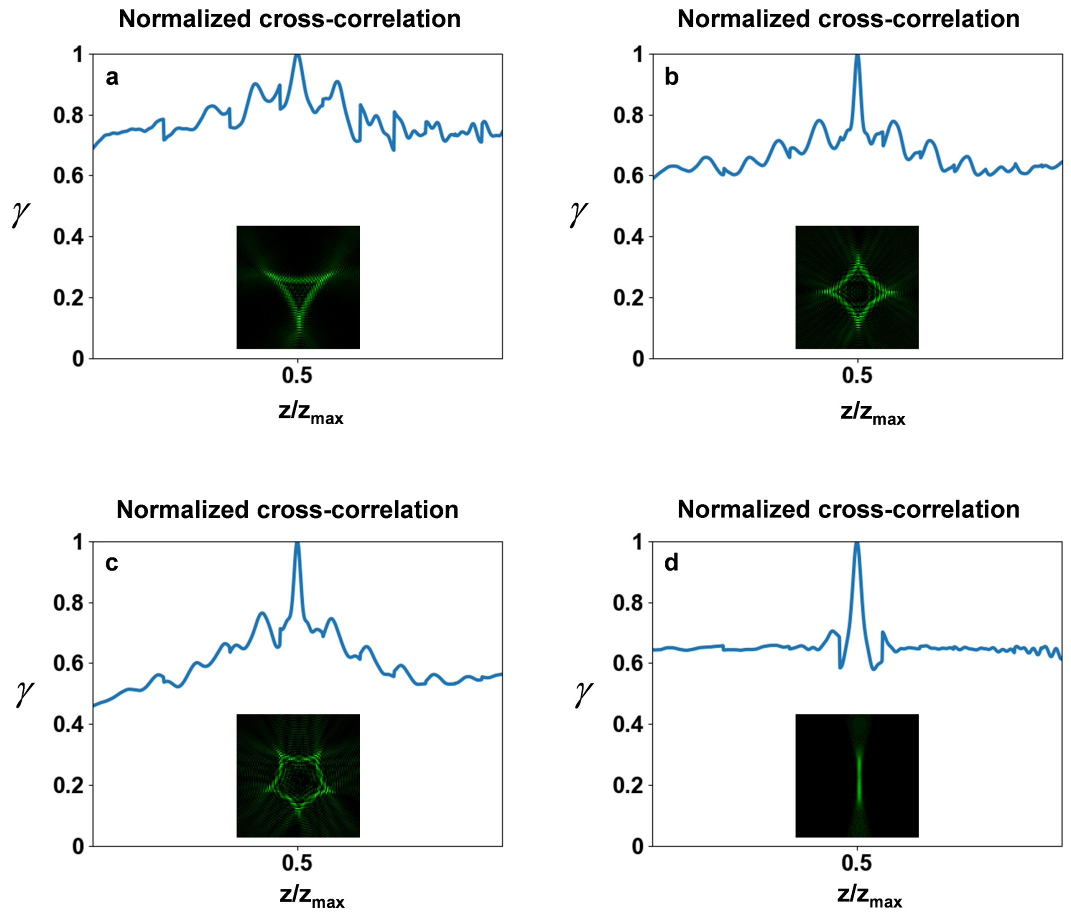

Fig. S16. Normalized cross-correlations as a demonstration of the invariance of deltoid, astroid, hypocycloid-5, and line segment caustics to obstructions.

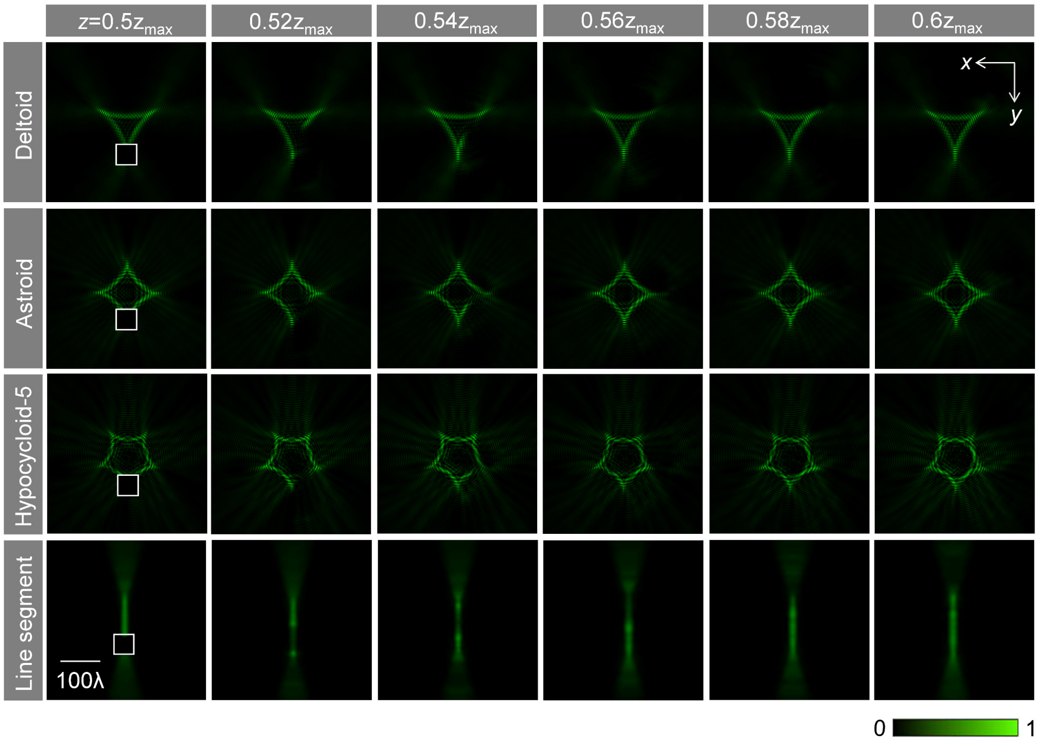

Fig. S17. The self-healing of the caustic structured light upon propagation. The blocked area is indicated by the white square box in the first column.

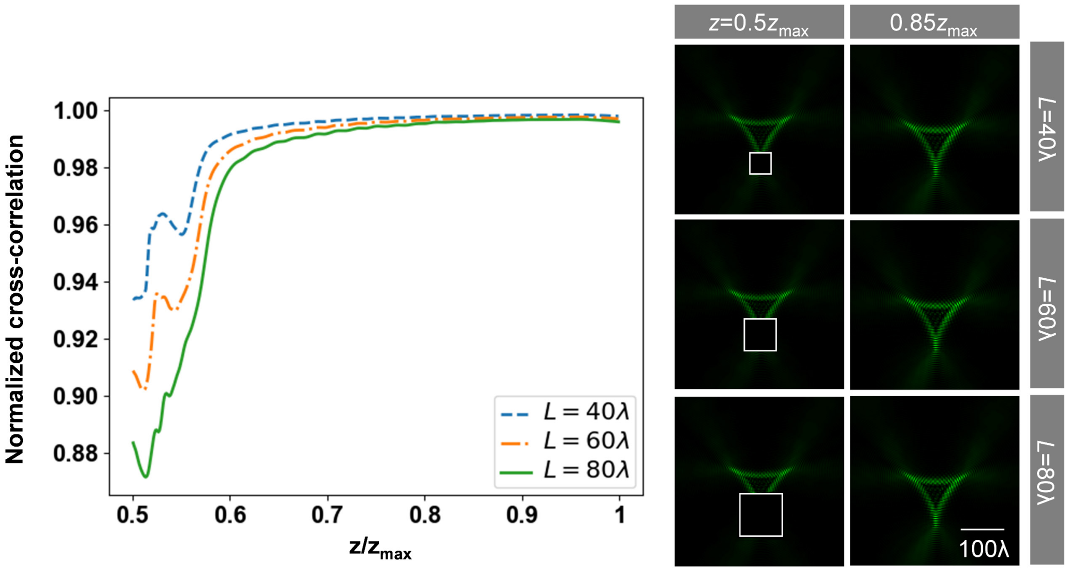

Fig. S18. The normalized cross-correlation coefficients between deltoid-shaped caustic beams blocked by obstacles with different sizes and those not affected by obstacles upon propagation. The blocked area is indicated by the white square box.

Supplementary Note 18. Intensity profiles under the condition of equal radii of Fourier rings.

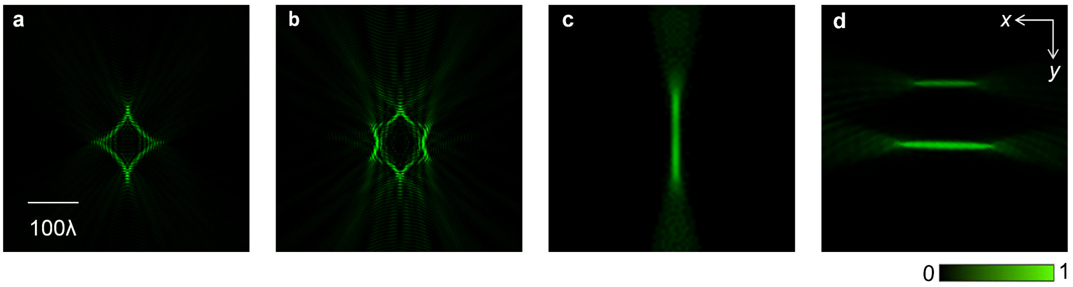

Fig. S19. Simulation results of Intensity profiles sculpted under the condition of equal radii of Fourier rings: deltoid (a), hypocycloid-6 (b), line (c), Chinese character for "two" (d).

Supplementary Note 19. Morphed caustics with a linear trajectory.

Arbitrary caustic engineering is achieved through the combined modulation in both longitudinal and transverse planes. If the propagation trajectory is preset as a straight line, the centers of the circles on the Fourier plane will remain fixed, and changing the information on different circles will lead to multiple interference. To circumvent this problem, we first design a curved propagation trajectory, which will cause the movement of the circle centers. Then, we distribute the Fourier information of different caustic patterns across various circles and compensate for the lateral displacement caused by the curved trajectory on the corresponding *x-y* plane, that is, an additional linear displacement for each caustic shape. In this way, we can realize the morphed caustic patterns along a linear trajectory. For illustration, we present the simulation results of caustic beams with shapes varying from deltoid to astroid, and then to hypocycloid with 5 cusps, along a linear trajectory, as shown in Fig. S20.

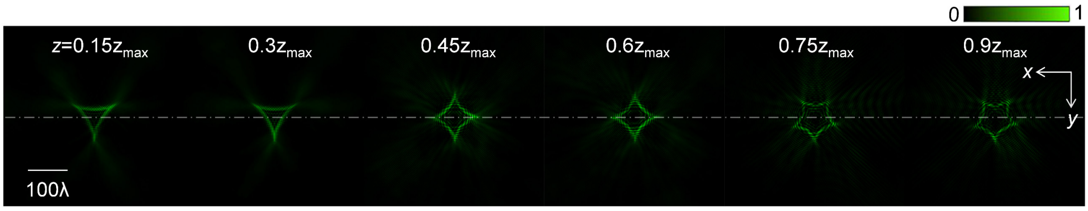

Fig. S20. Propagation dynamics of caustic beams with shapes varying from deltoid to astroid, and then to hypocycloid with 5 cusps, along a linear trajectory.

Supplementary Note 20. Derivation of the amplitude and phase of output from a meta-atom.

We utilize the Jones matrix to describe the amplitude and phase responses of a meta-atom. To begin, it is assumed that the incident light, coming from SiO_2 substrate side, carries left-handed circular polarization (LCP) $|L\rangle$:

$$|L\rangle = \frac{1}{2}\begin{bmatrix} 1 \\ i \end{bmatrix}. \quad (50)$$

The state of light as a function of propagation distance z through the meta-atom can be written as:

$$|\psi(z)\rangle = \Gamma(-\theta) M(z) \Gamma(\theta) |L\rangle, \quad (51)$$

with

$$M(z) = \begin{bmatrix} A_o(z)e^{i\varphi_o(z)} & 0 \\ 0 & A_e(z)e^{i\varphi_e(z)} \end{bmatrix}, \quad (52)$$

and a rotation matrix at an angle of θ

$$\Gamma(\theta) = \begin{bmatrix} \cos\theta & -\sin\theta \\ \sin\theta & \cos\theta \end{bmatrix}. \quad (53)$$

Here, $A_o(z)$, $A_e(z)$, and $\varphi_o(z)$, $\varphi_e(z)$ denote the amplitude and phase coefficients for the polarization along the long axis and short axis of the nanofin, respectively.

The Jones matrix of the meta-atom can be simplified to:

$$|\psi(z)\rangle = \frac{e^{i\frac{\varphi_o(z)+\varphi_e(z)}{2}}}{2\sqrt{2}} \begin{bmatrix} A_o(z)e^{i\varphi_\Delta}(1+e^{-i2\theta}) + A_e(z)e^{-i\varphi_\Delta}(1-e^{-i2\theta}) \\ iA_o(z)e^{i\varphi_\Delta}(1-e^{-i2\theta}) + iA_e(z)e^{-i\varphi_\Delta}(1+e^{-i2\theta}) \end{bmatrix}, \quad (54)$$

with

$$\varphi_\Delta = \frac{\varphi_o(z) - \varphi_e(z)}{2}. \quad (55)$$

The right-handed circular polarization (RCP) component of the light after a propagation distance H (the height of the meta-atom) is selected by the polarization filter, which can be represented as the inner product of $|R\rangle$ and $|\psi(H)\rangle$:

$$S = \langle R|\psi(H)\rangle = \frac{1}{2}\left[A_o(H)e^{i\varphi_o(H)} - A_e(H)e^{i\varphi_e(H)}\right]e^{-i2\theta}. \quad (56)$$

Here, we define that $t_l = \frac{1}{2}A_o(z)e^{i\varphi_o(H)}$ and $t_s = \frac{1}{2}A_e(z)e^{i\varphi_e(H)}$ are the S-parameters for the polarization along the long axis and short axis, respectively. Therefore, the amplitude and initial phase are determined as follows:

$$A_{\text{out}} = |t_l - t_s|, \quad (57)$$

and

$$\varphi_{\text{out}} = \arctan(t_l - t_s). \quad (58)$$

References

1. Airy, G. On the intensity of light in the neighbourhood of a caustic. *Trans. Camb. Philos. Soc.* **6**, 379 (1838).
2. Pearcey, T. XXXI. The structure of an electromagnetic field in the neighbourhood of a cusp of a caustic. *Lond. Edinb. Phil. Mag.* **37**, 311 (1946).
3. Berry, M. V. & Upstill, C. Catastrophe optics: morphologies of caustics and their diffraction patterns. *Prog. Opt.* **28**, 257-346 (1980).
4. Arnol'd, V. I. & Wassermann, G. S. *Catastrophe Theory* (Springer, Berlin, 1986).
5. Nye, J. F. *Natural Focusing and Fine Structure of Light* (IOP Publishing, Bristol,1999).
6. Siviloglou, G. A., Broky, J., Dogariu, A. & Christodoulides, D. N. Observation of accelerating Airy beams. *Phys. Rev. Lett.* **99**, 213901 (2007).
7. Ring, J. D. et al. Auto-focusing and self-healing of Pearcey beams. *Opt. Express* **20**, 18955-18966 (2012).
8. Zannotti, A., Diebel, F. & Denz, C. Dynamics of the optical swallowtail catastrophe. *Optica* **4**, 1157-1162 (2017).
9. Mamsch, C., Zannotti, A. & Denz, C. Embedding umbilic catastrophes in artificially designed caustic beams. *CLEO Europe*, EF_4_5 (2017).
10. Zannotti, A., Denz, C., Alonso, M. A., & Dennis, M. R. Shaping caustics into propagation-invariant light. *Nat. Commun.* **11**, 3597 (2020).
11. Baumgartl, J., Mazilu, M. & Dholakia, K. Optically mediated particle clearing using Airy wavepackets. *Nat. Photonics* **2**, 675-678 (2008).
12. Vettenburg, T. et al. Light-sheet microscopy using an Airy beam. *Nat. Methods* **11**,541-544 (2014).
13. Mathis, A. et al. Micromachining along a curve: femtosecond laser micromachining of curved profiles in diamond and silicon using accelerating beams. *Appl. Phys. Lett.* **101**, 0711101 (2012).
14. Zhang, J. C. et al. A 6G meta-device for 3D varifocal. *Sci. Adv.* **9**, eadf8478 (2023).
15. Zannotti, A. *Caustic Light in Nonlinear Photonic Media* (Springer International Publishing, 2020).
16. Born, M. & Wolf, E. *Principles of Optics* (Pergamon Press, Oxford, 1970).
17. Poston, T. & Stewart, I. *Catastrophe Theory and Its Applications* (Dover Publications Inc., Mineola,1996).
18. Berry, M.V. & Upstill, C. *Catastrophe Optics: Morphologies of Caustics and Their Diffraction patterns* (Progress Optic, Bristol, 1980).
19. Kravtsov, Yu. A. & Orlov, Yu. I. *Caustics, Catastrophes and Wave Fields* (Springer-Verlag, Berlin, 1999).
20. Whittaker, E. T. On the partial differential equations of mathematical physics. *Mathematische Ann.* **57**, 333-355 (1903).
21. Broky, J., Siviloglou, G. A. Dogariu, A. & Christodoulides, D. N. Self-healing properties of optical Airy beams. *Opt. Express* **16**, 12880-12891 (2008).
22. Gao, X.-Z. et al. Redistributing the energy flow of tightly focused ellipticity-variant vector optical fields. *Photon. Res*. **5**, 640-648 (2017).
23. Kaso, A. Computation of the normalized cross-correlation by fast Fourier transform. *PLoS ONE* **13**, e0203434(2018).
