# Supplementary material for: Arbitrary engineering of spatial caustics with 3D-printed metasurfaces
Source: Nat Commun. 2024 May 2;15:3719. doi: 10.1038/s41467-024-48026-5 (PMC11065864; doi:10.1038/s41467-024-48026-5)
Supplement: Supplementary file 3 — Description of Additional Supplementary Files [file 41467_2024_48026_MOESM3_ESM.pdf]

Description of Additional Supplementary Files

File Name: Supplementary Movie

Description: Fabrication of 3D-printed metasurfaces.
